# Operations on the Liver

**Published:** 1898-04-02

**Authors:** Leonard A. Bidwell

**Affiliations:** Senior Assistant-Surgeon to the Hospital


### Medical Progress and Hospital Clinics.

[*The Editor will be glad to receive offers of co-operation and contributions from members of the profession. All letters should be addressed to* The Editor, at the Office, 28 & 29, Southampton Street, Strand, London, W.C.]

### OPERATIONS ON THE LIVER.

A Postgraduate Lecture, delivered at the West London Hospital by Leonard A. Bidwell, F.R.C.S., Senior Assistant-Surgeon to the Hospital.

(*Continued from page* 447, *Vol. XXIII.*)

The two chief indications for operation on the gall-bladder are—(1) Repeated attacks of biliary colic, and (2) the presence of a tumour in the region of the gall-bladder. These may both be present or both absent in cases of gall-stones, but one of these two symptoms should be present before an operation is done. Other symptoms demanding operation are jaundice, with other signs of obstruction of the common bile duct; localised inflammation in the region of the gall-bladder, and acute peritonitis due to perforation.

Among the conditions which can be confounded with hepatic colic may be mentioned dyspepsia, cancer of the head of the pancreas, malignant disease of the pylorus, and movable kidney. The pain in gall-stone colic comes on some hours after food, whereas in dyspepsia it appears within half an hour, and so makes the diagnosis easy. In cancer of the head of the pancreas, the jaundice is persistent, the emaciation is marked, and the pain is less acute. In malignant disease of the pylorus the stomach is usually, but not always, dilated, and acute attacks of colic are not common unless the glands in the portal fissure are affected. In floating or cystic kidney Fremissen's test, as described before, will show us that the tumour is not connected with the liver.

In the following cases the symptoms so closely resembled gall-stone colic that an exploration of the gall-bladder was undertaken:—

Case VI.: A man, aged 54, was admitted into the West London Hospital, under Dr. Ball. For thirty years he had suffered from attacks of gnawing pain in the epigastric region; there was also an indefinite history of jaundice and biliary colic with enlargement of the liver. A hard mass, apparently connected with the liver, was felt in the region of the gall-bladder. There was slight jaundice, occasional vomiting, and pain after food. The abdomen was opened in the middle line above the umbilicus, and the gall-bladder and ducts found to be empty. The tumour felt was one surrounding the pyloric orifice, and, as it appeared not to be malignant, Loretta's operation was performed, and the patient made a complete recovery. He is alive and well now, nearly four years after the operation.

Case VII.: A woman, aged 45, was sent to me at the hospital by Dr. Kingsford, of Rushy Green. For about nine months she had suffered from attacks of hepatic colic, which were followed by jaundice that was rather persistent. The patient had lost flesh. A distinct tumour could be felt in the region of the gall-bladder. The abdomen was opened in the right linea semilunaris, the gall-bladder was a little distended. There was a large mass of malignant disease in the pylorus, and this was adherent to the under part of the liver. Enlarged glands could be felt in the portal fissure, which evidently caused the hepatic colic. As no radical operation was possible nothing further was done, and the wound was closed. The patient made a good recovery from the operation, and left the hospital three weeks later. The attacks of pain were less, and her general condition improved.

Case VIII.: A. P., aged 22, was sent to me at the West London Hospital by Dr. Cox, of Watford, with a history of gall-stone colic. On examination, a tumour, just below and continuous with the liver, could be felt. On operation the gall-bladder was found to be empty and normal, but the right kidney was movable, so nephorraphy was performed, and the patient had no further attacks of pain.

Case IX.: Mrs. T., aged 41, was admitted into the hospital on account of attacks of colic, commencing in the region of the gall-bladder, which were followed by jaundice. There was a tumour just below the liver. The abdomen was opened and the gall-bladder found to be normal. The tumour proved to be an enormously hypertrophied portion of omentum (about 2 in. thick), and the traction of this heavy mass upon the gall-bladder and pylorus had produced the symptoms. The patient was fitted with a special belt to support the abdomen, and obtained complete relief.

A tumour connected with the gall-bladder may consist either of gall-stones, fluid, or growth; the fluid may be either bile, mucus, or pus. A distended gall-bladder may exist without any stone, since the orifice of the cystic gut may be closed by inspissated mucus, by a kink, by traction, or by cicatricial contraction, due to previous inflammation. The tumour formed by the gall-bladder may be of large size, extending even to the brim of the pelvis; but its enlargement at first is in the line drawn from the top of the ninth rib to the umbilicus. It can be diagnosed from tumours not connected with the liver by Fremissen's test, which has already been described.

The operations which are performed for the gall-stone trouble are the following: (1) Cholecystotomy; (2) cholecystectomy; (3) cholecyst-enterostomy; (4) choledocho-lithotrity; (5) choledocho-lithotomy, or choledochotomy; (6) choledocho-enterostomy; (7) choledochectomy; (8) hepaticostomy. The names of these operations are rather formidable, and most of them are only extensions of the first-named.

**Cholecystotomy,** or opening the gall-bladder, is the operation which is most frequently performed, and is all that is required in most cases. It is indicated in the following conditions: (*a*) Empyema of the gall-bladder; (*b*) distension, with mucus; (*c*) gall-stones in the gall-bladder, or in the cystic duct; and (*d*) rupture or perforation. The operation is performed by making an incision 3 in. long just below the tumour (if present), or in the right linea semilunaris, commencing at the tip of the ninth rib. When the peritoneum is opened the gall-bladder is freed from adhesions and opened after sponges have been packed round it. The edges of the incision in the gall-bladder are held up with clamp forceps, and the gall-stones removed with a scoop and small lithotomy forceps; those in the cystic duct are cleared out in the same way. The edges of the gall-bladder are then united to the parietal peritoneum, and a drainage tube inserted through the gall-bladder into the cystic duct; the rest of the peritoneal wound is closed with silk sutures, and the skin and fascia united with silkworm gut. The drainage tube is removed in two or three days, and usually a large amount of bile escapes for from three to ten days, but the wound is generally healed within three weeks.

In the last series of published cases the mortality of the operation is stated to be 15 in 161 cases, or about 9·2 per cent.; that is probably a little higher than is now the case. Closure of the opening in the gall-bladder, the so-called ideal operation, is not to be recommended, since, if any obstruction to the flow of bile arise, extravasation into the peritoneal cavity will result.

*(To be continued.)*

